# MDM2 Drives Proteasome Inhibitor Resistance and Represents a TP53-Independent Therapeutic Vulnerability in Multiple Myeloma

**DOI:** 10.3390/cells15090831

**Published:** 2026-05-01

**Authors:** María Labrador, Sara Cozzubbo, Mariangela Porro, Michela Cumerlato, Cecilia Bandini, Elisabetta Mereu, Tina Paradzik, Benedetta Donati, Veronica Manicardi, Domenica Ronchetti, Mattia D’Agostino, Alessandra Larocca, Francesca Gay, Benedetto Bruno, Alessia Ciarrocchi, Andrew Chatr-Aryamontri, Antonino Neri, Eugenio Morelli, Roberto Piva

**Affiliations:** 1 Department of Molecular Biotechnology and Health Sciences, University of Turin, 10126 Turin, Italy; maria.labradorgranados@unito.it (M.L.); sara.cozzubbo@unito.it (S.C.); mariangela.porro@unito.it (M.P.); michela.cumerlato@unito.it (M.C.); cecilia.bandini@unito.it (C.B.); elisabetta.mereu@unito.it (E.M.); mattia.dagostino@unito.it (M.D.); alessandra.larocca@unito.it (A.L.); francesca.gay@unito.it (F.G.); benedetto.bruno@unito.it (B.B.); 2 Molecular Biotechnology Center (MBC) “Guido Tarone”, 10126 Turin, Italy; 3 Division of Physical Chemistry, Rudjer Boskovic Institute, 10000 Zagreb, Croatia; tina.paradzik@irb.hr; 4 Laboratory of Translational Research, Azienda USL-IRCCS di Reggio Emilia, 42122 Reggio Emilia, Italy; benedetta.donati@ausl.re.it (B.D.); veronica.manicardi@ausl.re.it (V.M.); alessia.ciarrocchi@ausl.re.it (A.C.); antonino.neri@ausl.re.it (A.N.); 5 Department of Oncology and Hemato-Oncology, University of Milan, 20122 Milan, Italy; domenica.ronchetti@unimi.it; 6 Division of Hematology, AOU Città della Salute e della Scienza di Torino, University of Turin, 10126 Turin, Italy; 7 ChemoGenix CRISPR Screening Platform, Institute for Research in Immunology and Cancer, Université de Montréal, Montreal, QC H3T 1J4, Canada; andrew.chatr-aryamontri@umontreal.ca; 8 Candiolo Cancer Institute, FPO-IRCCS, 10060 Candiolo, Italy; eugenio.morelli@ircc.it; 9 Department of Oncology, University of Turin, 10124 Turin, Italy

**Keywords:** MDM2, multiple myeloma, proteasome inhibitors, *TP53*, drug resistance, synthetic lethality

## Abstract

**Highlights:**

**What are the main findings?**

- Functional screens identify MDM2 as a driver of carfilzomib resistance in multiple myeloma.
- MDM2 inhibition synergizes with proteasome inhibitors independently of *TP53* status.

**What are the implications of the main findings?**

- MDM2 is a therapeutically actionable vulnerability in proteasome inhibitor-resistant and *TP53*-deficient multiple myeloma.
- Dual MDM2/proteasome targeting induces synthetic lethality via cell-cycle arrest and impaired DNA damage response.

**Abstract:**

Proteasome inhibitors (PIs) are central to multiple myeloma (MM) therapy; however, resistance remains a major clinical challenge, particularly in relapsed/refractory disease. To identify functional mediators of carfilzomib (CFZ) resistance, we performed complementary gain-of-function CRISPR activation and pharmacological screening approaches. These unbiased strategies converged on the E3 ubiquitin ligase MDM2 as a modulator of PI response. MDM2 transactivation enhanced MM cell survival and accelerated recovery following CFZ exposure, supporting a causal role in proteotoxic stress tolerance. Pharmacologic inhibition of MDM2 with NVP-CGM097 synergized with CFZ across multiple PI-sensitive and PI-resistant MM cell lines, irrespective of *TP53* status. Mechanistically, MDM2 inhibition induced p21 upregulation, cell-cycle arrest, and reduced c-MYC expression, accompanied by impaired activation of DNA damage response mediators. Genetic silencing of MDM2 phenocopied these effects and increased CFZ sensitivity. Importantly, the combination retained efficacy in MM–stromal co-culture models and in primary patient samples, including cases harboring del(17p), while sparing normal peripheral blood mononuclear cells. Collectively, these findings identify MDM2 as a functional driver of PI resistance and support combined MDM2 and proteasome inhibition as a rational therapeutic strategy in MM, including *TP53*-deficient contexts.

## 1. Introduction

Multiple myeloma (MM) is a plasma cell malignancy arising in the bone marrow, characterized by high genetic complexity and intraclonal heterogeneity [1]. The introduction of proteasome inhibitors (PIs) into MM treatment regimens significantly improved patient outcomes and today remains a cornerstone of therapy in both newly diagnosed and relapse settings [2,3]. Proteasome inhibitors target the 20S core of the 26S proteasome, a central component of one of the two major protein degradative pathways—the ubiquitin proteasome system (UPS) [4]. Malignant plasma cells are highly dependent on efficient protein turnover to sustain proliferation and immunoglobulin synthesis, which renders them particularly vulnerable to proteasome inhibition [5]. Under deficient proteasome capacity, MM cells show decreased protein synthesis and increased protein workload, which exacerbate endoplasmic reticulum stress and activate the unfolded protein response, leading to apoptosis [6]. Despite the efficacy of PIs, MM cells can develop resistance through multiple mechanisms, including *PSMB5* mutations, deregulation of proteasomal subunits, an increase in protein degradation by the autophagy–lysosomal pathway, and activation of DNA damage repair responses [7,8]. Although these mechanisms have been extensively characterized in preclinical models, their clinical relevance remains difficult to interpret. Because PIs are administered as part of combination regimens, disentangling drug-specific resistance phenotypes in patients is particularly challenging. Consistently, studies profiling relapsed/refractory MM (RRMM) have identified recurrent molecular signatures associated with PI resistance, such as activation of the unfolded protein response (UPR), cell-cycle dysregulation, stress adaptation, and enhanced DNA repair [9,10]. In recent years, novel immunotherapies such as BCMA- or GPRC5D-targeting bispecific antibodies and BCMA CAR T-cell products, as well as targeted agents including the exportin-1 inhibitor selinexor, the BCL-2 inhibitor venetoclax, and the JAK2 inhibitor ruxolitinib, have demonstrated encouraging activity in the relapse setting [11]. However, for patients who are refractory or ultimately relapse after these last therapeutic options, the identification of new vulnerabilities and combinatorial strategies remains an urgent unmet need.

Mouse double minute 2 homolog (MDM2) is an E3 ubiquitin ligase best known for binding and ubiquitinating p53, promoting its nuclear export, and targeting it for proteasomal degradation, thereby suppressing p53-transcriptional activity [12]. In addition to its canonical role as a negative regulator of p53, MDM2 exerts multiple p53-independent functions through interactions with a range of cellular substrates, including transcription factors such as E2F1, cell-cycle regulators such as p21, and members of the p53 family such as p73. Moreover, MDM2 has been implicated in the regulation of DNA damage responses through interactions with components of the MRN complex, including NBS1 [13,14].

Previous studies demonstrated that MDM2 promotes myeloma cell proliferation and survival [15,16]. Small-molecule inhibitors that disrupt MDM2-p53 interaction, such as nutlins, have shown anti-myeloma activity by inducing apoptosis [17]. However, MDM2-p53 inhibitors have shown limited activity in the context of *TP53* inactivation. This may be particularly relevant in MM, where *TP53* mutations or deletions are associated with resistance to standard therapy and poor prognosis [18]. Moreover, the clinical efficacy of MDM2 inhibitors as a monotherapy has been modest, and hematopoietic toxicities are common side effects, suggesting that combination strategies may provide a more effective therapeutic approach.

In this study, through complementary genetic gain-of-function and pharmacologic loss-of-function screening approaches, we identify MDM2 as a functional modulator of carfilzomib (CFZ) resistance in MM. By evaluating the therapeutic efficacy of MDM2 inhibitor NVP-CGM097 in MM cell lines and patient samples, our results indicate that combined MDM2 and proteasome inhibition represents a promising therapeutic strategy, including in the context of *TP53* inactivation.

## 2. Materials and Methods

### 2.1. Cell Culture Conditions and Reagents

Human multiple myeloma (MM) cell lines (KMM-1, U266, RPMI 8226, KMS-18, KMS-27, KMS-28, KMS-11, OPM-2, and AMO-1) and the human stromal cell line HS-5 were obtained from DSMZ (German Collection of Microorganisms and Cell Cultures, Braunschweig, Germany) or ATCC (American Type Culture Collection, Manassas, VA, USA). Proteasome inhibitor (PI)-resistant cell lines (KMM-1 PIR, U266 PIR, AMO-1 CFZ-R, AMO-1 BTZ-R, RPMI 8226 CFZ-R, and RPMI 8226 BTZ-R) were generated in-house by stepwise exposure with increasing concentrations of BTZ or CFZ, as previously described [19,20], or kindly provided by Dr. Lenka Besse (Department of Oncology and Hematology, Kantonsspital St Gallen, Switzerland) [21,22]. All cell lines were authenticated by DNA fingerprinting using the GenePrint system (Promega, Madison, WI, USA). Cells were maintained in RPMI 1640 supplemented with 10% fetal bovine serum, L-glutamine, penicillin, and streptomycin (Euroclone, Milan, Italy), at 37 °C in a humidified atmosphere with 5% CO_2_. HEK293T cells obtained from DSMZ were cultured in DMEM supplemented as detailed before. Carfilzomib (#HY-10455), bortezomib (#HY-10227), and NVP-CGM097 (#HY-15954) were obtained from MedChemExpress (Stockholm, Sweden). Blasticidin S hydrochloride (#SC204655) was obtained from Santa Cruz Biotechnology (Dallas, TX, USA). Puromycin dihydrochloride (#P833) was obtained from Sigma-Aldrich (St. Louis, MO, USA). Hygromycin (#ant-hg-1) was obtained from Invivo Gen (Toulouse, France).

### 2.2. Lentivirus Production and In Vitro Transduction

Lentiviral particles were produced in low-passage HEK293T cells using Effectene Transfection reagent (Qiagen, Hilden, Germany) and packaging vectors psPAX2 and pMD2G (Addgene #12260 and #12259). Viral supernatants harvested in the next two days were filtered through 0.22 μm filters and concentrated 100× using Lenti-X™ Concentrator (Takara Bio, San Jose, CA, USA). MM cell lines (1 × 10^5^/mL) were transduced with virus aliquots in the presence of 8 μg/mL polybrene. Antibiotic selection was initiated 24 h after infection and cell transduction efficiency was evaluated in the following 48–96 h.

### 2.3. CRISPR Activation (CRISPRa) Screening

A human CRISPR/Cas9 synergistic activator mediator (SAM) library (Addgene #100000007), a gift from F. Zhang, was amplified by electroporation as previously described [23] and DNA was extracted using the Endofree Plasmid Maxi kit (Qiagen, Hilden, Germany). AMO-1 cells were sequentially transduced with the SAM components to stably express dCas9-VP64 and MS2-p65-HSF1 (CVPH). A total of 1.2 × 10^8^ AMO-1 CVPH cells were transduced with the lentiviral sgRNA library at a multiplicity of infection of 0.3 and a ratio > 500 cells/sgRNA. Two separate infections were carried out. Following puromycin selection and recovery, cells were expanded. At the beginning of the screens, 4 × 10^7^ cells (500 cells/sgRNA) were collected as baseline controls and 4 × 10^7^ cells/condition were treated with CFZ or DMSO (<0.001%) for 21–35 days. At the end of the screening, 4 × 10^7^ cells/condition were harvested for DNA extraction and NGS. Genomic DNA was extracted, PCR amplified, and deep sequenced to examine sgRNA representation, as previously described [24]. Raw FASTQ files were processed with cutadapt for the trimming of sequencing adapters and reads were mapped to sgRNA sequences in the library. The MAGeCK-MLE algorithm was applied to analyze differential sgRNA abundance between CFZ- and DMSO-treated populations.

### 2.4. Drug Screening

A 320-inhibitor library (L3500, Selleck Chemicals) targeting 123 key proteins involved in diverse signaling pathways was screened in U266 PIR cells in combination with a sublethal CFZ treatment [20,25]. Growth rate (GR) was assessed using Cell Titer-Glo luminescence assay (Promega, Madison, WI, USA) at 0 and 72 h post-treatment. The combined drug effect was evaluated by Excess over Bliss (EOB) analysis using the formula

EOB = (1 − GR_combination) − (1 − GR_CFZ) − (1 − GR_drug) + (1 − GR_CFZ) x (1 − GR_drug),

where positive EOB values indicate synergism and negative EOB values indicate antagonism [26].

### 2.5. MDM2 sgRNA and shRNA Constructs

For MDM2 transactivation, sgRNA oligos were selected from the SAM library and cloned into lenti-sgRNA(MS2)_puro backbone (#73795). The vector backbone was digested using BsmBI (Thermo Scientific, Waltham, MA, USA), gel extracted with the QIAquick Gel Extraction Kit (Qiagen, Hilden, Germany), dephosphorylated with AP (Roche, Basel, Switzerland) and purified using the Monarch PCR&DNA Cleanup kit (New England Biolabs, Ipswich, MA, USA). Equal amounts of complementary sgRNA oligonucleotides (100 μM) were mixed with T4 DNA ligase buffer (Roche, Basel, Switzerland) and T4 polynucleotide kinase (PNK) enzyme (New England Biolabs, Ipswich, MA, USA) for annealing and phosphorylation. Annealed oligos were diluted 1:200 and ligated overnight into the BsmBI-digested lentiviral vector using T4 ligase (Roche). The ligation was transformed into One Shot Stbl3 chemically competent *E. coli* (Invitrogen, Waltham, MA, USA). Plasmid DNA was extracted using the PureYield Plasmid Miniprep System (Promega, Madison, WI, USA) and Sanger sequenced before lentivirus production. For MDM2 silencing, TRCN0000355727 shRNA clone was purchased from Sigma-Aldrich and transformed into One Shot Stbl3 chemically competent *E. coli* (Invitrogen, Waltham, MA, USA).

### 2.6. DNA Sequencing

Genomic DNA of AMO-1 and RPMI 8226 cell lines was extracted using the Quick-DNA Miniprep kit (Zymo Research, Irvine, CA, USA). Exon 8 of the TP53 gene was analyzed by PCR amplification and Sanger sequenced. Primer sequences are available in the Supplementary Materials.

### 2.7. Cell Viability and Cell-Cycle Analysis

Cell viability was measured by flow cytometry (FACS) after staining with tetramethylrhodamine methyl ester (TMRM; Molecular Probes, Eugene, OR, USA) or propidium iodide (Miltenyi Biotec, Bergisch Gladbach, Germany), according to the manufacturer’s instructions. Cell-cycle analysis was performed by FACS in cells treated with RNase (0.14 mg/mL) and stained with propidium iodide (28.57 μg/mL). Data were acquired using a BD FACSCelestaTM or BD AccuriTM cytofluorimeter and processed with FACSDiva 8.0 software (BD Biosciences, San Jose, CA, USA).

### 2.8. MM/BMSCs 2D Co-Culture

HS-5 stromal cells and RFP-positive RPMI 8226 cells were mixed at a concentration of 5 × 10^4^ cells/mL and 1 × 10^5^ cells/mL, respectively, in a 24-well plate. The co-cultures were treated with 5 nM CFZ, 5 μM NVP-CGM097, or a combination of the two drugs. Every three–four days, both the suspension and adherent cells were collected for FACS analysis and re-plated into new wells. The percentage of RFP^+^ cells was measured by FACS.

### 2.9. Cells Isolated from Healthy Donors and MM Patients

Peripheral blood mononuclear cells (PBMCs) from healthy donors were kindly provided by the local Blood Bank (Città Della Salute e della Scienza Hospital, Turin). Bone marrow cells were obtained from routine BM aspirates of myeloma patients (Città Della Salute e della Scienza Hospital, Turin). PBMCs were collected from buffy coat by Ficoll-Hypaque density gradient separation, suspended in complete RPMI 1640, and seeded (5 × 10^5^ cells/mL). BM cells were isolated by Ficoll-Hypaque gradient centrifugation, according to Miltenyi’s protocol. CD138^+^ cells were detected by FACS analysis by using anti-CD138-APC (Clone 44F9, Miltenyi). Samples containing more than 5% CD138^+^ myeloma cells were seeded (2.5 × 10^5^ cells/mL) in complete RPMI 1640 and treated with CFZ and NVP-CGM097. Treatments and cytogenic and clinical features of patients are described in the Supplementary Materials.

### 2.10. Purification of Total RNA and Reverse Transcription-Quantitative Polymerase Chain Reaction (RT-qPCR)

Total RNA was extracted using the RNeasy mini kit (Qiagen) according to the manufacturer’s instructions, then treated with RQ1 RNase-free DNase (Promega, Madison, WI, USA). cDNA was reverse transcribed from total RNA using OneScript Plus cDNA synthesis kit (Applied Biological Materials, Richmond, BC, Canada), following the manufacturer’s instructions. Quantitative PCR reactions were performed in 384-well plates with a Thermal iCycler (Bio-Rad, Hercules, CA, USA) using the Bio-Rad iQ SYBR Green Supermix according to the manufacturer’s instructions. The PCR cycling conditions were as follows: 95 °C for 5 min followed by 40 cycles at 94 °C for 10 s and 60 °C for 30 s. The oligonucleotide primer pairs used for RT-qPCR were designed with PrimerBLAST (Primer3 version 2.5.0) (http://www.ncbi.nlm.nih.gov/tools/primer-blast/ accessed on 30 April 2022) and are listed in the Supplementary Materials. All PCR assays were performed in triplicate, and the average Ct (cycles to threshold) was used for the comparative Ct method. Amplification specificity was confirmed by analysis of the melting curve of the PCR products. Quantification of GAPDH levels served as an endogenous control.

### 2.11. Western Blotting

Protein extracts were prepared using Lysis Buffer containing 20 mM Tris-HCl (pH 7.4), 150 mM NaCl, 5 mM EDTA, 1% Triton X-100, 1 mM PMSF, 10 mM NaF, 1 mM Na_3_VO_4_, and Protease Inhibitor Cocktail (Roche). Total protein concentrations were measured using the Bio-Rad DC protein assay kit (Bio-Rad, Hercules, CA, USA). Equal amounts of protein lysates were resolved by SDS-PAGE, transferred to nitrocellulose membrane, blocked for 1 h at RT with 5% low-fat milk in phosphate-buffered saline + 0.1% Tween 20 (PBST), and probed overnight with primary antibodies diluted in 5% BSA + 0.01% NaN_3_ in PBST at 4 °C. Membranes were then incubated with the secondary antibody diluted 1:10,000 in 5% low-fat milk in PBST for 1 h. Immune complexes were detected using Immobilon Western Chemiluminescent HRP Substrate (Merck, Rahway, NJ, USA) using Chemidoc Instrument (Bio-Rad, Hercules, CA, USA). The antibodies used in the study are listed in the Supplementary Materials.

### 2.12. Statistical Analysis

Statistical analyses were performed with GraphPad Prism 5.01 (GraphPad Software Inc., San Diego, CA, USA). Statistical significance was determined by Student’s *t* test and one-way ANOVA analysis as indicated throughout the manuscript. Differences were considered significant when the *p* value was <0.05 (*), <0.01 (**), <0.001 (***), or <0.0001 (****).

## 3. Results

### 3.1. Functional Screenings Converge on MDM2 as a Modulator of Proteasome Inhibitor Resistance in Multiple Myeloma

**Figure 1:**
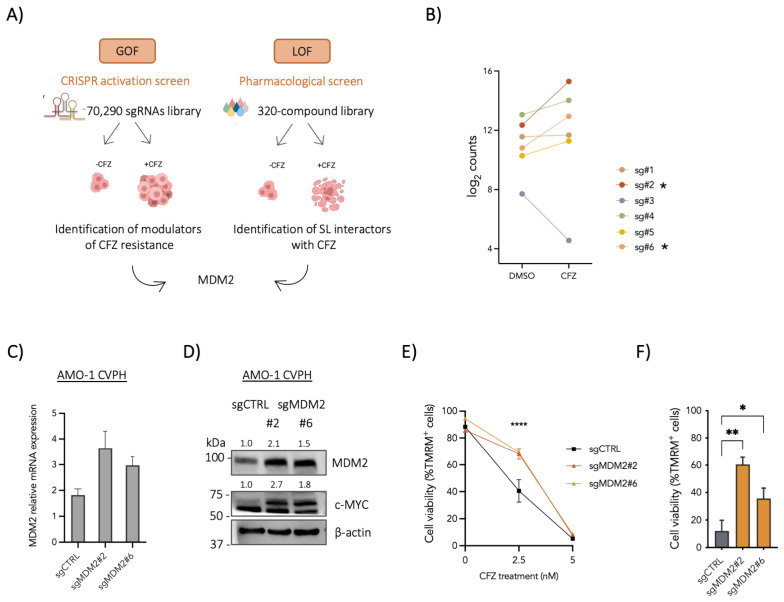
Complementary functional screenings identify MDM2 as a modulator of carfilzomib (CFZ) resistance in multiple myeloma. (**A**) Schematic overview of the complementary screening strategies. A gain-of-function (GOF) CRISPR activation (CRISPRa) screen was performed in PI-sensitive AMO-1 CVPH cells treated with CFZ or DMSO to identify modulators of CFZ resistance. In parallel, a loss-of-function (LOF) pharmacological screen using a 320-compound library was conducted to identify compounds exhibiting synthetic lethality with CFZ. Both approaches converged on MDM2. (**B**) Log_2_-normalized read counts of sgRNAs targeting *MDM2* at the end of the CRISPRa screen (T_end). sgRNA enrichment is shown relative to the mean read counts in DMSO- or CFZ-treated conditions at T_end. The most enriched sgRNAs selected for individual validation are indicated by asterisks. (**C**–**F**) AMO-1 CVPH cells were transduced with two independent sgRNAs targeting *MDM2* or a non-targeting control sgRNA (sgCTRL). (**C**,**D**) MDM2 transactivation was confirmed at the mRNA and protein levels by RT–qPCR and immunoblotting, respectively. Band quantification values are indicated above each band (band intensities were normalized to β-actin and subsequently to the sgCTRL control). (**E**) Cells were subsequently treated with CFZ, and cell viability was assessed 72 h post-treatment by flow cytometry after staining with tetramethylrhodamine methyl ester (TMRM). (**F**) Recovery following treatment with 5 nM CFZ was evaluated after 7 days as above. Data represent mean ± SD of three independent experiments. Immunoblots are representative of three independent experiments. Statistical significance was determined by one-way ANOVA (* *p* < 0.05; ** *p* < 0.01; **** *p* < 0.0001).

To identify novel regulators of carfilzomib (CFZ) resistance and uncover potential therapeutic vulnerabilities in multiple myeloma (MM), we employed complementary functional screening approaches. As a gain-of-function strategy, we conducted a genome-wide CRISPR activation (CRISPRa) screen in the proteasome inhibitor (PI)-sensitive AMO-1 cell line. AMO-1 cells stably expressing the CRISPR-dCas9 SAM activation system (AMO-1 CVPH) were transduced with a pooled sgRNA library and cultured in the presence of CFZ or DMSO vehicle for 21–35 days. Next-generation sequencing (NGS) was performed at baseline and at the end of the treatment to assess changes in sgRNA representation. Gene-level enrichment analysis using MAGeCK-MLE identified multiple candidate modulators of CFZ response, including both previously described and novel genes associated with proteasome inhibitor resistance. Among these, the E3 ubiquitin ligase *MDM2* emerged as one of the most significantly enriched in CFZ-treated populations compared with controls (Figure 1A,B). In parallel, an independent pharmacological screen performed in the PI-resistant U266 cell line [20,25,27] identified the MDM2 inhibitor NVP-CGM097 as synergistic with CFZ (Figure 1A), further implicating MDM2 in modulating CFZ response.

To functionally validate the role of MDM2 in CFZ resistance, AMO-1 CVPH cells were transduced with two independent sgRNAs targeting *MDM2* or a non-targeting control sgRNA (sgCTRL). MDM2 transactivation resulted in increased MDM2 mRNA and protein expression (Figure 1C,D). Upon CFZ exposure, MDM2-transactivating cells displayed increased viability at sublethal CFZ concentrations compared with control cells (Figure 1E). Moreover, these cells demonstrated a faster recovery following lethal CFZ treatment (Figure 1F), indicating that MDM2 transactivation promotes survival under CFZ-induced proteotoxic stress.

Consistent with a previously reported positive regulatory interaction between MDM2 and MYC, whereby MDM2 stabilizes MYC mRNA and enhances its translation [28], we observed increased MYC protein levels following MDM2 transactivation in AMO-1 CVPH cells (Figure 1D). Together, these findings identify MDM2 as a functional mediator of proteasome inhibitor resistance in MM and support a model in which MDM2 contributes to CFZ tolerance, at least in part, through enhanced MYC expression.

### 3.2. Therapeutic Potential and Molecular Mechanisms Underlying NVP-CGM097/PI Combination

**Figure 2:**
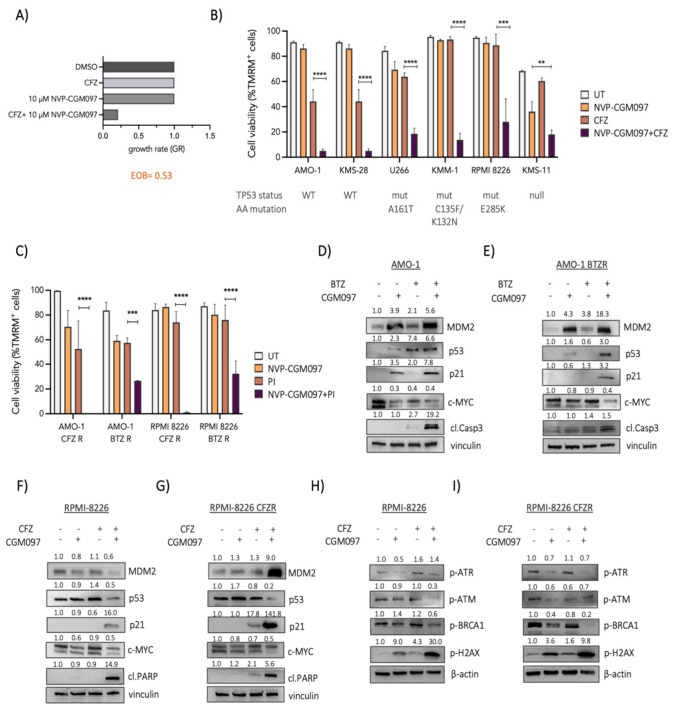
Pharmacologic inhibition of MDM2 with NVP-CGM097 synergizes with PIs in MM cells independently of *TP53* status. (**A**) Growth rate (GR) analysis of the PI-resistant U266 PIR cell line treated with NVP-CGM097, CFZ, or a combination, as identified in the pharmacological screen. Excess over Bliss (EOB) score indicates synergistic interaction. (**B**) Validation of the combinatorial effect of NVP-CGM097 and CFZ in a panel of PI-sensitive MM cell lines with distinct *TP53* status. Cell viability was assessed by flow cytometry as a percentage of TMRM-positive cells at 48 h or 72 h post-treatment. (**C**) Four PI-resistant MM cell lines were treated with NVP-CGM097, BTZ or CFZ, either as single agents or in combination. Cell viability was measured by TMRM staining 72 h post-treatment. Data represent mean ± SD of three independent experiments. Statistical significance was determined by one-way ANOVA (** *p* < 0.01; *** *p* < 0.001; **** *p* < 0.0001). (**D**–**I**) AMO-1 (*TP53* WT), AMO-1 BTZR, RPMI-8226 (*TP53* mutant), and RPMI-8226 CFZR cells were treated with NVP-CGM097 and PIs (BTZ or CFZ) as single agents or in combination. Protein expression of MDM2, p53, p21, c-MYC, cleaved PARP (cPARP), and DNA damage response markers (p-ATR, p-ATM, p-BRCA1, and p-H2AX) was analyzed by immunoblotting 24 h post-treatment. Vinculin or β-actin was used as a loading control. Band quantification values are indicated above each band (band intensities were normalized to loading controls and subsequently to the DMSO-treated condition). Immunoblots are representative of three independent experiments.

To validate and further characterize the therapeutic interaction between MDM2 inhibition and proteasome blockade, we examined the combinatorial effects of NVP-CGM097 and CFZ in MM models. Combined treatment with 20 nM CFZ and 10 μM NVP-CGM097 in the PI-resistant cell line U266 PIR significantly reduced cell growth rate compared with either single agent, with a positive Excess over Bliss (EOB) score (>0.5) consistent with a synergistic interaction (Figure 2A). To extend these findings beyond a single model, we treated a panel of six PI-sensitive and four PI-resistant MM cell lines with sublethal doses of NVP-CGM097, a PI (CFZ or BTZ), or a combination. Across all cell lines tested, the combination consistently and significantly decreased cell viability compared with single-agent treatments, irrespective of *TP53* mutational status (Figure 2B,C). Synergistic interactions were further supported by Bliss score analysis in multiple models (Figure S1). Importantly, the preservation of synergy in *TP53*-mutant models suggests that the therapeutic interaction between NVP-CGM097 and proteasome inhibitors is not strictly dependent on canonical p53 signaling. These findings indicate that MDM2 may contribute to PI response through additional p53-independent mechanisms.

To further investigate the mechanisms underlying NVP-CGM097/CFZ cytotoxicity, we selected a *TP53* wild-type (AMO-1) and a *TP53*-mutant (RPMI 8226) MM cell line. RPMI 8226 cells harbor a homozygous *TP53* missense mutation (p.E285K), previously characterized as a loss-of-function (LOF) alteration (Figure S2). Western blot analysis revealed that in *TP53* wild-type AMO-1 cells, NVP-CGM097 treatment induced accumulation of MDM2, p53, and p21, accompanied by a reduction in c-MYC protein levels (Figure 2D,E). In contrast, in *TP53*-mutant RPMI 8226 cells, MDM2 accumulation was predominantly observed upon combination treatment in the PI-resistant cells (Figure 2F,G). Basal p53 expression remained unchanged; however, p53 levels decreased following combination treatment. Similarly to AMO-1 cells, NVP-CGM097 reduced c-MYC expression, and combination therapy induced a marked upregulation of p21 (Figure 2F,G). The increase in p21 was consistent with G1 cell-cycle arrest induced by the combinatorial treatment (Figure S3). Beyond the canonical MDM2-p53 axis, MDM2 has been reported to directly bind p21 and promote its proteasomal degradation [29]. Accordingly, p21 accumulation in *TP53*-mutant cells likely reflects relief from MDM2-mediated destabilization rather than transcriptional activation via p53. To explore additional mechanisms contributing to the response in *TP53*-mutated cells, we examined the expression of proteins involved in DNA damage signaling and repair. Combination treatment resulted in increased levels of phosphorylated H2AX, indicating persistent DNA damage (Figure 2H,I). Notably, NVP-CGM097 reduced ATR phosphorylation and, particularly under the combination treatment, decreased phosphorylated BRCA1 levels, suggesting impaired activation of DNA damage response pathways (Figure 2H,I). To further corroborate the functional consequences of MDM2 inhibition, MDM2 was silenced through constitutive expression of specific shRNA. MDM2 knock-down resulted in p21 induction, promoted G1 cell-cycle arrest, and enhanced sensitivity to CFZ treatment, phenocopying the effects observed with pharmacological inhibition (Figure S4).

Collectively, these findings support a role for MDM2 inhibition in potentiating PI-induced cytotoxicity through both p53-dependent and p53-independent mechanisms.

### 3.3. Preclinical Efficacy and Safety Profile of NVP-CGM097/CFZ Combination

**Figure 3:**
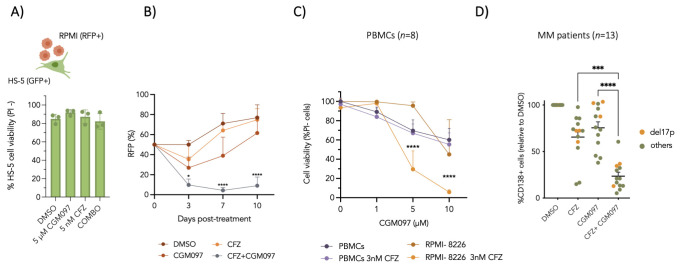
The NVP-CGM097/CFZ combination is effective in 2D MM/BMSC co-cultures and primary MM patient samples while sparing normal cells. (**A**,**B**) RPMI-8226 cells stably expressing RFP (RFP^+^) and HS-5 bone marrow stromal cells expressing GFP (GFP^+^) were cultured either alone or in co-culture and treated with 5 µM NVP-CGM097, 5 nM CFZ, or a combination. HS-5 cell viability was assessed by propidium iodide (PI) staining 72 h post-treatment (**A**). Viability of RPMI-8226 cells in co-culture was evaluated by flow cytometry as the percentage of RFP^+^ cells over a 10-day period (**B**). Data represent mean ± SD of five independent experiments. Statistically significant differences between the combination treatment and CGM097 are represented. (**C**) Peripheral blood mononuclear cells (PBMCs) isolated from healthy donors (*n* = 8) were treated with NVP-CGM097, 3 nM CFZ, or a combination. RPMI-8226 cells treated under the same conditions were included as a tumor control. Cell viability was determined by flow cytometry following PI staining 72 h post-treatment. (**D**) Primary CD138^+^ plasma cells isolated from bone marrow aspirates of MM patients (*n* = 13) were treated with NVP-CGM097, CFZ, or a combination. The drug concentrations used in these studies are specified in Table S2. Cell viability was assessed 72 h post-treatment by flow cytometry using CD138-APC staining and expressed relative to DMSO-treated controls. Samples harboring del(17p) are indicated separately. Statistical significance was determined by one-way ANOVA (* *p* < 0.5; *** *p* < 0.001; **** *p* < 0.0001). In the graphs, NVP-CGM097 is abbreviated as CGM097.

To assess the translational relevance of the NVP-CGM097/CFZ combination, we examined its efficacy in more physiologically representative models of MM. Given the established role of the bone marrow (BM) microenvironment in sustaining MM proliferation and conferring drug resistance, we co-cultured RFP-expressing RPMI 8226 cells with GFP-positive HS-5 bone marrow stromal cells. The combinatorial treatment selectively reduced the viability of MM cells while sparing stromal HS-5 cells, and this effect was sustained over a 10-day observation period (Figure 3A,B). These findings indicate that the NVP-CGM097/CFZ combination remains effective despite microenvironment-derived protective signals. 

To assess potential off-target toxicity, peripheral blood mononuclear cells (PBMCs) from healthy donors (*n* = 8) were exposed to increasing concentrations of NVP-CGM097 in the presence or absence of CFZ. Only modest reductions in PBMC viability were observed at the highest NVP-CGM097 concentrations tested, supporting a therapeutic window between malignant and non-malignant cells (Figure 3C).

We next evaluated the combination in primary MM patient samples (*n* = 13). Clinical and cytogenetic characteristics of the patient cohort, including prior treatments, are summarized in Table S3. Treatment with NVP-CGM097/CFZ resulted in a significant reduction in the proportion of CD138^+^ plasma cells compared with single agents, indicating enhanced cytotoxicity in patient-derived tumor cells (Figure 3D). Notably, the combinatorial effect was preserved in samples harboring del(17p), a cytogenetic alteration commonly associated with *TP53* loss and adverse prognosis, suggesting that therapeutic efficacy is not strictly dependent on intact p53 signaling.

Collectively, these findings indicate that the NVP-CGM097/CFZ combination exhibits selective anti-myeloma activity, limited toxicity toward normal PBMCs, and preserved efficacy in high-risk primary MM samples, supporting its translation potential.

## 4. Discussion

Using complementary unbiased screening approaches, including a genome-wide CRISPR activation screen and an independent pharmacological synergy screen, we identified the E3 ubiquitin ligase MDM2 as a functional determinant of CFZ resistance and a therapeutically targetable vulnerability in MM. The CRISPRa screen identified multiple candidate modulators of CFZ response, underscoring the robustness of the approach. MDM2 was prioritized for further investigation based on its strong enrichment and independent validation through pharmacological screening. Additional candidate regulators are currently under investigation in ongoing studies. Our data demonstrate that enforced MDM2 activation enhances survival under CFZ-induced stress and accelerates post-treatment recovery, thereby establishing a causal role for MDM2 in PI resistance.

Given its role as a negative regulator of p53, MDM2 has emerged as a therapeutic target across multiple malignancies, including MM. Genetic silencing of MDM2 induces cell-cycle arrest and apoptosis in MM cells [15,16]. MDM2 overexpression has been reported in MM and associated with advanced disease stage, drug resistance, and poor prognosis [15,16,28]. In particular, Faruq et al. demonstrated that MDM2 contributes to drug resistance through reciprocal regulation of c-MYC and both p53-dependent and p53-independent pathways [28]. MDM2 inhibitors disrupt the MDM2–p53 interaction by occupying the hydrophobic pocket that binds critical p53 residues (Phe19, Trp23, and Leu26) [30]. Several small-molecule inhibitors—including nutlin derivatives, MI-63, and AMG-232—have demonstrated preclinical anti-myeloma activity, predominantly in *TP53* wild-type contexts [17,31]. However, accumulating evidence indicates that MDM2 inhibition can also exert cytotoxic effects independently of *TP53* status [28,32].

Here, we extend this concept by showing, through unbiased functional screening and validation across multiple models, that MDM2 inhibition potentiates PI-induced cytotoxicity irrespective of *TP53* mutational status. Importantly, the synergistic interaction was achieved using sublethal doses of each agent, supporting a synthetic vulnerability rather than additive toxicity. While Faruq et al. established a critical role for the MDM2–MYC axis in mediating drug resistance and demonstrated enhanced sensitivity to bortezomib using the dual MDM2/XIAP inhibitor MX69, our study provides complementary evidence derived from unbiased functional screening and extends these observations to carfilzomib-based combinations across multiple PI-sensitive and PI-resistant models using a selective MDM2 inhibitor. MX69 promotes MDM2 degradation and inhibits XIAP translation, whereas NVP-CGM097 was designed to disrupt the p53-MDM2 interaction [33]. However, inhibition of the MDM2 hydrophobic pocket may induce conformational changes that alter its interactions with additional protein partners. It is known that MDM2 can regulate p21 stability [29,34] and modulate cell-cycle progression through interactions with the Rb/E2F axis [35]. Consistent with these reports, we observed induction of p21 and cell-cycle arrest following NVP-CGM097 treatment even in *TP53*-mutant models. In addition, our data support the previously proposed MDM2–c-MYC regulatory axis in PI-resistance [28], although the precise causal contribution of c-MYC downregulation to the observed synergistic interaction remains to be formally established.

In addition to these pathways, MDM2 has been shown to regulate multiple p53-independent substrates, including members of the p53 family such as p73, transcription factors such as E2F1, and proteins involved in DNA damage response pathways [13,14]. Notably, the interaction between MDM2 and p73 is complex and context-dependent, involving modulation of transcriptional activity and, in certain settings, protein stability rather than a consistent degradative mechanism [36,37,38]. These observations highlight the broader regulatory role of MDM2 beyond p53 and suggest that coordinated modulation of multiple p53-independent pathways may contribute to the cellular response to combined MDM2 and proteasome inhibition.

Here, we found that the NVP-CGM097/CFZ combination altered DNA damage response (DDR) signaling. Combination treatment increased γH2AX levels while reducing phosphorylation of key DDR mediators, suggesting impaired repair capacity. While these findings suggest impairment of DNA damage response signaling, the temporal relationship between DDR inhibition and apoptosis remains to be determined, and time-course studies will be required to distinguish primary from secondary effects. MDM2 has been reported to interact with the MRN complex and modulate double-strand break repair [35,39]. Nutlin-induced MDM2 accumulation has also been shown to suppress MRN-dependent repair, sensitizing cells to genotoxic stress [40]. Our findings are consistent with a model in which MDM2 inhibition exacerbates PI-induced stress by compromising DNA repair mechanisms, contributing to synthetic lethality. In line with these observations, recent work in other tumor models has shown that proteasome inhibition enhances the efficacy of MDM2-targeting strategies through activation of stress response pathways, including synergistic effects with CFZ [41].

Importantly, the therapeutic relevance of our findings extends beyond cell line systems. The NVP-CGM097/CFZ combination retained activity in MM–bone marrow stromal cell (BMSC) co-culture models. Ex vivo experiments using primary MM bone marrow samples confirmed robust cytotoxicity, including in samples harboring high-risk cytogenetic abnormalities such as 1q amplification and 17p deletion. Although the combination retained activity in samples harboring high-risk cytogenetic abnormalities such as del(17p), these observations should be interpreted with caution, given the limited size of the patient cohort, and require validation in larger, clinically annotated studies. Since most primary samples were derived from smoldering or newly diagnosed patients, validation in relapsed/refractory MM cohorts will be important to determine its clinical relevance in relapsed/refractory disease.

Clinically, MDM2 inhibitors have already entered early-phase trials in MM. AMG-232 has been evaluated in combination with carfilzomib and dexamethasone in relapsed/refractory disease [42], and idasanutlin has been tested with ixazomib-based regimens in patients with del(17p) (NCT02633059). NVP-CGM097 and the second-generation inhibitor HDM201 (siremadlin) have demonstrated activity in *TP53* wild-type solid tumors and hematologic malignancies [43,44]. Our data suggest that combining MDM2 inhibition with proteasome blockade may extend therapeutic benefit to *TP53*-mutant MM. However, the clinical development of MDM2 inhibitors has been limited by modest single-agent activity, and their incorporation into current MM treatment regimens will require careful evaluation in the context of increasingly complex combination therapies. In addition, the concentrations used in vitro were selected to model sublethal conditions for synergy assessment and may not directly correspond to clinically achievable exposures.

Besides small-molecule inhibitors, strategies such as PROTAC degraders are being explored to target MDM2. The MDM2 degraders MD-224 and MD-265 have demonstrated rapid MDM2 degradation and potent antitumor activity in preclinical models [45,46,47,48]. Whether MDM2 degraders provide superior efficacy over classical pocket inhibitors in *TP53*-mutant MM warrants investigation.

In summary, although prior studies have suggested limited efficacy of MDM2 inhibitors in *TP53*-mutant settings, our data demonstrate that combining MDM2 inhibition with proteasome inhibitors induces robust cytotoxicity irrespective of *TP53* status. Notably, this synergistic effect was achieved using sublethal single-agent doses while sparing normal peripheral blood mononuclear cells, supporting a favorable therapeutic window. However, PBMCs represent mature hematopoietic cells and do not fully recapitulate the complexity of the bone marrow niche. Given the known hematologic toxicities associated with MDM2 inhibitors [49], further studies will be required to assess the impact of this combination on hematopoietic progenitor cells and bone marrow homeostasis. While our findings are supported by multiple in vitro and ex vivo models, including microenvironment co-culture systems and primary patient samples, the absence of in vivo validation represents a limitation of the present study and will need to be addressed in future work using xenograft or PDX mouse models. Furthermore, a deeper mechanistic investigation of p53-independent pathways is warranted. Collectively, our results establish MDM2 as a functional mediator of proteasome inhibitor resistance and provide a strong preclinical rationale for combining MDM2 inhibitors with PIs, including in *TP53*-mutant MM.

## 5. Conclusions

This study identifies MDM2 as a functional mediator of proteasome inhibitor resistance in MM and demonstrates that its pharmacological inhibition enhances the efficacy of proteasome inhibitors independently of *TP53* status. Importantly, this synthetic interaction is preserved in microenvironment-supported models and in primary MM patient samples, supporting its translational relevance. These findings provide a rationale for further in vivo validation and clinical evaluation of combined MDM2 and proteasome inhibition, including in high-risk and *TP53*-deficient disease.

## Data Availability

The original contributions presented in this study are included in the article/Supplementary Materials. Further inquiries can be directed to the corresponding author.

## Supplementary Materials

The following supporting information can be downloaded at: https://www.mdpi.com/article/10.3390/cells15090831/s1, Figure S1: Bliss synergy score of NVP-CGM097/CFZ combinations; Figure S2: g.7673767C>T *TP53* mutation in RPMI-8226 cell line; Figure S3: Cell-cycle analysis of NVP-CGM097/CFZ combinations; Figure S4: MDM2 silencing in AMO-1 cells; Table S1: Drug concentrations used for cell treatments in MM cell lines.; Table S2: Drug concentrations used in ex-vivo assays with bone-marrow derived mononuclear cells (BMMCs); Table S3: Cytogenetic and clinical features of patients included in ex vivo assays; Table S4: Primer sequences used in the present study; Table S5: Antibodies used in the present study.

## Abbreviations

The following abbreviations are used in this manuscript:

ATF4	Activating transcription factor 4
ATM	Ataxia telangiectasia mutated
ATR	Ataxia telangiectasia and Rad3-related protein
BLC-2	B-cell lymphoma 2
BM	Bone marrow
BMSCs	Bone marrow stromal cells
BRCA1	Breast cancer type 1 susceptibility protein
BTZ	Bortezomib
BTZ-R	Bortezomib-resistant
CFZ	Carfilzomib
CFZ-R	CFZ-resistant
CRISPR	Clustered regularly interspaced short palindromic repeat
CRISPRa	CRISPR activation
Ct	Cycle threshold
CTRL	Control
CVPH	dCas9-VP64-MS2-p65-HSF1
DDR	DNA damage response
DMEM	Dulbecco’s modified Eagle medium
DMSO	Dimethyl sulfoxide
EOB	Excess over Bliss
FACS	Fluorescence-activated cell sorting
GFP	Green fluorescent protein
GOF	Gain of function
GR	Growth rate
JAK2	Janus kinase 2
LOF	Loss of function
MDM2	Mouse double minute 2 homolog
MM	Multiple myeloma
MRN	MRE11-RAD50-NBS1 complex
NDMM	Newly diagnosed multiple myeloma
NGS	Next generation sequencing
PBMCs	Peripheral blood mononuclear cells
PDX	Patient-derived xenograft
PI	Propidium iodide
PIR	Proteasome inhibitor resistance
PIs	Proteasome inhibitors
PROTAC	Proteolysis-targeting chimera
PSMB5	Proteasome subunit beta 5
RB	Retinoblastoma protein
RFP	Red fluorescent protein
RT-qPCR	Reverse transcription quantitative polymerase chain reaction
RRMM	Relapse refractory multiple myeloma
SAM	Synergistic activator mediator
sgRNA	Single-guide RNA
shRNA	Short hairpin RNA
SMM	Smoldering multiple myeloma
TMRM	Tetramethylrhodamine methyl ester
TP53	Tumor protein p53 gene
UPR	Unfolded protein response
UPS	Ubiquitin proteasome system
XIAP	X-linked inhibitor of apoptosis protein
γH2AX	Phosphorylated histone H2AX
