# Supplementary material for: MDM2 Drives Proteasome Inhibitor Resistance and Represents a TP53-Independent Therapeutic Vulnerability in Multiple Myeloma
Source: Cells. 2026 May 1;15(9):831. doi: 10.3390/cells15090831 (PMC13162650; doi:10.3390/cells15090831)
Supplement: Supplementary file 1 [file cells-15-00831-s001.zip › cells-4267478-supplementary.pdf]

Supplementary Materials

MDM2 Drives Proteasome Inhibitor Resistance and Represents a TP53-Independent Therapeutic Vulnerability in Multiple Myeloma

María Labrador[1,2], Sara Cozzubbo[1,2], Mariangela Porro[1,2], Michela Cumerlato[1,2], Cecilia Bandini[1,2], Elisabetta Mereu[1,2], Tina Paradzik[3], Benedetta Donati[4], Veronica Manicardi[4], Domenica Ronchetti[5], Mattia D'Agostino[1,6], Alessandra Larocca[1,6], Francesca Gay[1,6], Benedetto Bruno[1,6], Alessia Ciarrocchi[4], Andrew Chatr-Aryamontri[7,8], Antonino Neri[5], Eugenio Morelli[9,10], Roberto Piva[1,2,6]*

[1]Department of Molecular Biotechnology and Health Sciences, University of Turin, 10126 Turin, Italy; [2]Molecular Biotechnology Center (MBC) "Guido Tarone", 10126 Turin, Italy; [3]Department of Physical Chemistry, Rudjer Boskovic Institute, 10000 Zagreb, Croatia; [4]Laboratory of Translational Research, Azienda USL-IRCCS di Reggio Emilia, 42122 Reggio Emilia, Italy; [5]Department of Oncology and Hemato-Oncology, University of Milano, 20122 Milano, Italy; [6] Division of Hematology, AOU Città della Salute e della Scienza di Torino, University of Turin, 10124 Turin, Italy; [7]Institute for Research in Immunology and Cancer, Université de Montréal, Montreal, QC, Canada; [8]ChemoGenix CRISPR Screening Platform, Institute for Research in Immunology and Cancer, Université de Montréal, Montreal, QC, Canada; [9]Candiolo Cancer Institute, FPO-IRCCS, 10060 Candiolo, Italy; [10]Department of Oncology, University of Turin, 10124 Turin, Italy.
*Correspondence: roberto.piva@unito.it.

Supplementary Figures

Figure S1. Bliss synergy score of NVP-CGM097/CFZ combinations.
Figure S2. g.7673767C>T *TP53* mutation in RPMI-8226 cell line.
Figure S3. Cell cycle analysis of NVP-CGM097/CFZ combinations.
Figure S4. MDM2 silencing in AMO-1 cells.

Supplementary Tables

Table S1. Drug concentrations used for cell treatments in MM cell lines.
Table S2. Drug concentrations used in ex-vivo assays with marrow–derived mononuclear cells (BMMCs).
Table S3. Cytogenetic and clinical features of patients included in ex vivo assays.
Table S4. Primer sequences used in the present study.
Table S4. Antibodies used in the present study.

Supplementary Figures

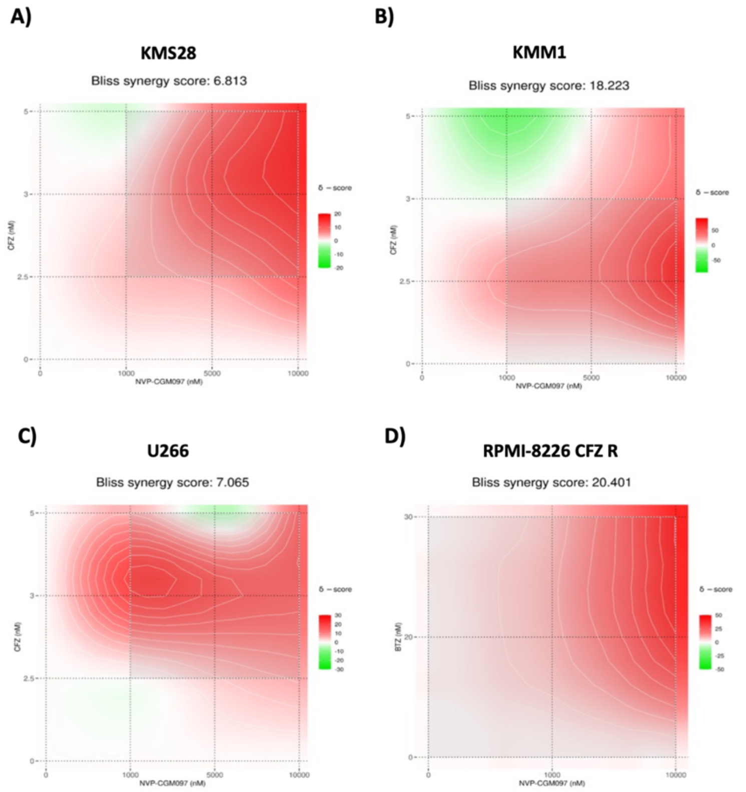

Figure S1. Bliss synergy score of NVP-CGM097/CFZ combinations. 2D dose-response matrices showing the combinatorial effects of NVP-CGM097 and CFZ in KMS28, KMM1, U266, and RPMI 8226 CFZR cell lines. Cell viability was assessed by TMRM staining and analyzed by flow cytometry 72 hours post-treatment. Bliss Synergy (BS) scores were calculated using the SynergyFinder+ tool (www.synergyfinder.org).

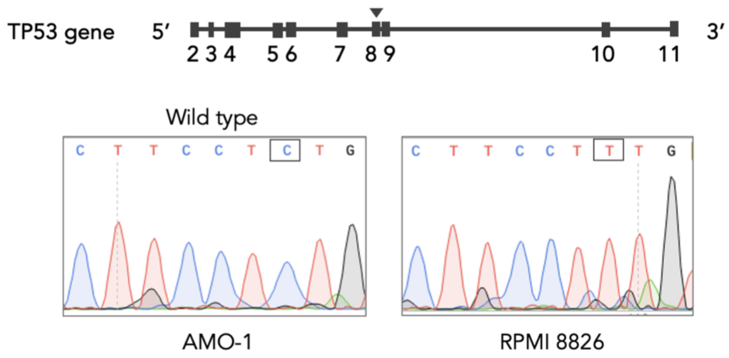

Figure S2. g.7673767C>T *TP53* mutation in RPMI-8226 cell line. Graphical design of *TP53* gene sequence. Nucleotide sequence corresponding to exon 8 shows the g.7673767 C>T mutation present in the RPMI 8226 cell line compared with the wild-type sequence in AMO-1.

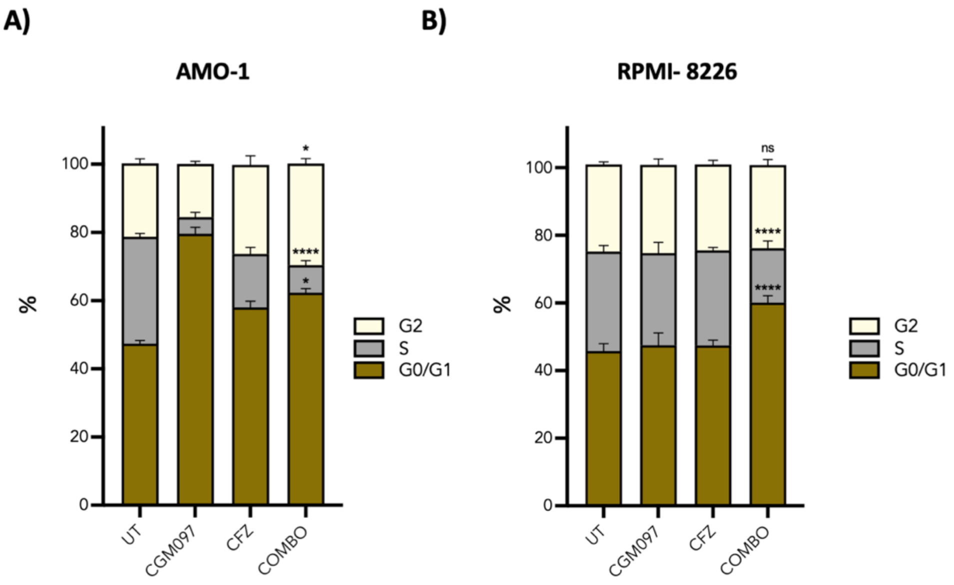

Figure S3. Cell cycle analysis of NVP-CGM097/CFZ combinations. AMO-1 and RPMI 8226 cells were treated with NVP-CGM097 and CFZ as indicated in Table S1. Cell cycle was measured by PI staining-flow cytometry 24 hpt. Yellow, grey, and white bars represent G0/G1, S, and G2/M fractions respectively. Data represent the mean ± SD of three technical replicates from one of three independent biological experiments performed. Statistical significance was determined by one-way ANOVA; significant differences between CFZ and NVP-CGM097+CFZ treatment are indicated (*p < 0.05; ****p < 0.0001). In the graphs, NVP-CGM097 is abbreviated as CGM097.

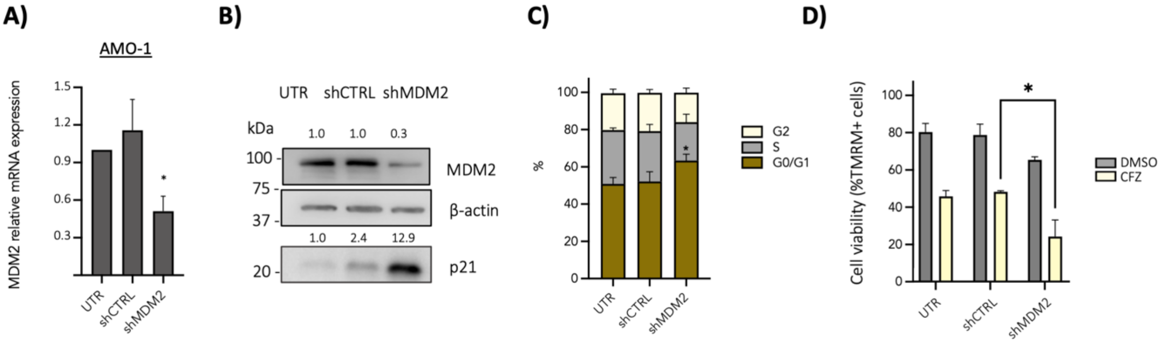

Figure S4. MDM2 silencing in AMO-1 cells. AMO-1 cells were transduced with a *MDM2*-targeting shRNA or a non-targeting control (shCTRL). **(a,b)** MDM2 silencing was confirmed 96 hours post-infection by (a) RT–qPCR (b) and immunoblotting. Band quantification values are indicated above each band (band intensities were normalized to β-actin and subsequently to the UTR) **(c)** Cell cycle was measured by PI staining-flow cytometry 96h post transduction. **(d)** Cell viability was measured by TMRM staining 72 hours post-treatment with 3nM CFZ. Data represent mean ± SD of three independent experiments. Statistical significance was determined by one-way ANOVA (*p < 0.05). Indicated statistical differences refer to (a) shCTRL versus shMDM2; (c) UTR versus shMDM2.

Supplementary Tables

Table S1. Drug concentrations used for cell treatments in MM cell lines.

Cell line	NVP-CGM097 (µM)	CFZ (nM)	BTZ (nM)
AMO-1	1	3	
KMS-28	10	1.25	
U266	10	3	
KMM-1	10	2.5	
RPMI 8226	5	3	
KMS-11	2	2.5	
AMO-1 CFZR	1.5	200	
AMO-1 BTZR	2		90
RPMI-8226 CFZR	10	80	
RPMI-8226 BTZR	10		150

Table S2. Drug concentrations used in ex-vivo assays with marrow–derived mononuclear cells (BMMCs).

Patient (PT)	NVP-CGM097 (µM)	CFZ (nM)
8	5	1.25
9	5	1.25
18	1	1.25
30	5	3
31	1	2.5
34	5	3
36	1	3
43	1	2.5
47	1	1.25
51	1	0.625
54	1	1.25
56	1	0.625
60	2	1.25

Table S3. Cytogenetic and clinical features of patients included in ex vivo assays.

PATIENT No.	Year of birth	SMM vs NDMM vs RRMM	Del(17p)	T(4;14)	T(14;16)	Gain/Amp (1q)	Del(1p)	T(11;14)	Del(13q)	Disease isotype	ISS at diagnosis	No. Prior lines	Drug exposure	Drug refractoriness
8	1944	NDMM	neg	neg	neg	NV	NV	NV	NV	FLC k	N/A	0	N/A	N/A
9	1959	SMM	neg	neg	neg	NV	NV	NV	NV	IgG l	N/A	0	N/A	N/A
18	1961	RRMM	pos	neg	neg	pos	neg	pos	pos	FLC k	2	1	BTZ, THAL,MEL, LENA	N/A
30	N/A	NDMM	pos	neg	neg	pos	neg	NV	NV	IgG k	1	0	N/A	N/A
31	1954	NDMM	neg	neg	pos	pos	neg	NV	NV	IgG k	3	0	N/A	N/A
34	1950	SMM	neg	neg	neg	pos	neg	neg	pos	IgA k	N/A	0	N/A	N/A
36	1935	SMM	pos	pos	neg	pos	neg	neg	pos	IgG l	N/A	0	N/A	N/A
43	1947	NDMM	neg	pos	NV	NV	NV	NV	pos	IgG l	3	0	N/A	N/A
47	1942	NDMM	neg	pos	neg	neg	neg	neg	pos	IgG k	2	0	N/A	N/A
52	1954	RRMM	pos	N/A	N/A	pos	neg	N/A	pos	FLC l	3	1	THAL	LENA
54	1935	NDMM	neg	pos	NV	pos	neg	NV	pos	IgG l	3	0	N/A	N/A
56	1945	SMM	neg	neg	neg	pos	neg	neg	pos	IgG k	N/A	0	N/A	N/A
60	1945	NDMM	neg	neg	neg	pos	neg	neg	pos	IgGk	1	0	N/A	N/A

Table S4. Primer sequences used in the present study.

Oligos	Sequence (5’-3’)
sgRNA oligo NT1 top	CACCGCTGAAAAAGGAAGGAGTTGA
sgRNA oligo NT1 bot	AAACTCAACTCCTTCCTTTTTCAGC
sgRNA oligo MDM2#2 top	CACCGGGCTCGGCCGCACCACCTC
sgRNA oligo MDM2#2 bot	AAACGAGGTGGTGCGGCCGAGCCC
sgRNA oligo MDM2#6 top	CACCGTAGTCTGGGCGGGATTGGGC
sgRNA oligo MDM2#6 bot	AAACGCCCAATCCCGCCCAGACTAC
MDM2_1098 Fw	GCCTGTAGTGAAGAAGGACAAGA
MDM2_1156 Rv	TCTCCCCTGCCTGATACACA
p53 exon 8 sense 1	TATCCTGAGTAGTGGTAATC
p53 exon 8 antisense 2	AAGTGAATCTGAGGCATAAC
shRNA Control oligo top	CCGGCAACAAGATGAAGAGCACCAACTCGAGTTGGTGCTCTTCATCTTGTTGTTTTT
shRNA MDM2 (TRCN0000355727)	CCGGCTCTCGACTCAGAAGATTATACTCGAGTATAATCTTCTGAGTCGAGAGTTTTTG

Table S5. Antibodies used in the present study.

Antibody	Species	Source
β-actin (clone C4)	mouse	#MAB1501, Merck Millipore
Vinculin	mouse	#SAB4200080, Sigma
α-tubulin (clone B512)	mouse	# T5168, Sigma
MDM2 (D1V2Z)	rabbit	#86934S, Cell Signaling Technology
Cleaved PARP-1	rabbit	#5625, Cell Signaling Technology
Cleaved caspase-3	rabbit	#9664, Cell Signaling Technology
p-Histone H2A.X (S139) (20E3)	rabbit	#9718, Cell Signaling Technology
p-ATR (S428)	rabbit	#2853, Cell Signaling Technology
p-ATM (S1981) (D6H9)	rabbit	#5883, Cell Signaling Technology
p-BRCA1 (S1524)	rabbit	#9009, Cell Signaling Technology
p21	mouse	#610241, BD Biosciences
p53 (1C12)	mouse	#2524, Cell Signaling Technology
c-MYC (D84C12) XP ®	rabbit	#5605, Cell Signaling Technology
